# A reference genome for the eastern bettong ( *Bettongia gaimardi*)

**DOI:** 10.12688/f1000research.157851.2

**Published:** 2025-01-27

**Authors:** Luke W Silver, Richard J Edwards, Linda Neaves, Adrian D. Manning, Carolyn J Hogg, Sam Banks

**Affiliations:** 1 Australian Research Council Centre of Excellence for Innovations in Peptide and Protein Science, The University of Sydney, Camperdown, NSW, 2006, Australia; 2 The University of Sydney School of Life and Environmental Sciences, Camperdown, New South Wales, 2006, Australia; 3 Minderoo OceanOmics Centre at UWA, The University of Western Australia Oceans Institute, Crawley, Western Australia, 6009, Australia; 4 Evolution and Ecology Research Centre, University of New South Wales School of Biotechnology and Biomolecular Sciences, Kensington, New South Wales, 2033, Australia; 5 Australian National University Fenner School of Environment and Society, Acton, Australian Capital Territory, 2601, Australia; 6 Charles Darwin University Research Institute for the Environment and Livelihoods, Casuarina, Northern Territory, 0909, Australia

**Keywords:** Genome annotation, reference genome, bettong, marsupial

## Abstract

The eastern or Tasmanian bettong ( *Bettongia gaimardi*) is one of four extant bettong species and is listed as ‘Near Threatened’ by the IUCN. We sequenced short read data on the 10x system to generate a reference genome 3.46Gb in size and contig N50 of 87.36Kb and scaffold N50 of 2.93Mb. Additionally, we used GeMoMa to provide and accompanying annotation for the reference genome. The generation of a reference genome for the eastern bettong provides a vital resource for the conservation of the species.

## Introduction

The eastern or Tasmanian bettong ( *Bettongia gaimardi*) is a small nocturnal Australian marsupial in the potoroid family and is considered an important ecosystem engineer due to its habit of digging and feeding on fungi ( Munro et al. 2019, Ross et al. 2019a, 2019b). Eastern bettongs were once widespread across south-eastern Australia but are reported to have gone extinct on mainland Australia around the 1920’s due to predation from introduced carnivores and land clearing ( Short 1998). Eastern bettongs are now confined to the eastern half of Tasmania where they are listed as ‘Near Threatened’ by the IUCN Red List ( Burbidge et al. 2016).

Australia has experienced the highest extinction rate of mammals on any continent over the past 200 years, accounting for 28% of the world’s mammal extinctions since the year 1600 ( McKenzie et al. 2007). Nationwide, a number of reintroduction programs are being implemented for the conservation of locally-extinct mammals, typically in the ‘critical weight range’ of 35 – 5500g ( Burbidge and McKenzie 1989). In the case of the eastern bettong, the species was reintroduced from the state of Tasmania to two fenced reserves in the Australian Capital Territory (ACT) between 2011 and 2012 ( Batson et al. 2016).

The generation of a reference genome will provide a valuable resource in the management of the two reintroduced populations of eastern bettongs and contribute to the global effort to sequence all eukaryotic life on Earth ( Lewin et al. 2022). To generate a reference genome, we sequenced DNA with 10x Genomics short reads and used GeMoMa to produce a genome annotation.

## Methods

### Sample collection and DNA/RNA extraction and sequencing

We used muscle tissue from a deceased male pouch young ( *B. gaimardi*) individual collected from Mulligan’s Flat Woodland Sanctuary during population monitoring in 2014 and was frozen immediately after collection. The Sample was collected under Australian National University Animal Experimentation Ethics Committee ethics protocol A2011/017. DNA extraction used the QIAGEN Genomic Tips kit (Qiagen Catalogue # 10223) yielding 170ng/μl of DNA. Sequencing used the KAPA HyperPrep PCR free library kits (Roche Catalogue # KK8503) and two lanes of HiSeq Xten 150 bp PE sequencing (Illumina) at the Ramaciotti Centre for Genomics (UNSW, Sydney). Additional sequencing using the 10X Chromium Genomics library prep with >50 kb size selection was performed using 2 lanes of HiSeq Xten (150 bp PE) (Illumina). The sample was accessioned to the Australia Museum Accession number AM M.56404.

### Genome assembly

Raw 10x data was assembled using Supernova v2.0.0 (RRID:SCR_016756) ( Weisenfeld et al. 2017) with default settings and pseudohaplotype output. Haplotypes were tidied and filtered using Diploidocus v0.3.0 (RRID:SCR_021231) ( Stuart et al. 2022), which filtered redundant and 100% unresolved scaffolds, and assigned the longest version of each scaffold to haplotype 1 [bettong.v1.0]. Scaffolds flagged as diploid (identical in both haplotypes) were then added back to haplotype 2 and each haplotype ran through Telociraptor v0.9.0 ( https://github.com/slimsuite/telociraptor) to length-sort and rename scaffolds after modifying the ends to reveal telomeres, where appropriate [bettong.v1.1]. Scaffolds identified by Tiara v1.0.3 ( Karlicki et al. 2022) as being from archaea, bacteria, prokarya or organelle were filtered, along with scaffolds flagged for exclusion or review by FCS-GX [bettong.v1.2] ( Astashyn et al. 2023).

Completeness was estimated using Benchmarking Universal Single-Copy Orthologues (BUSCO, RRID:SCR_015008) v5.4.3 ( Simao et al. 2015) using the mammalia_odb10 (n:9226) and vertebrata_odb10 datasets (n:3354).

Repetitive elements of the genome were identified, classified and masked using a Pawsey Supercomputing Centre Nimbus cloud machine (256GB RAM, 64 vCPU, 3 TB storage) by building a database using RepeatModeler v2.0.1 (RRID:SCR_015027) ( Flynn et al. 2020) with default settings; repeats were then masked using RepeatMasker v4.0.9 (RRID:SCR_012954) ( Smit et al. 2013-2015).

### Genome annotation

A homology-based annotation was created independently for each haplotype using GeMoMa v1.9 (RRID:SCR_012954) ( Keilwagen et al. 2019) using the annotation from nine Ensembl mammalian genomes (cow [ *Bos taurus*], human [ *Homo sapiens*], opossum [ *Monodelphis domestica*], mouse [ *Mus musculus*], Tammar wallaby [ *Macropus eugenii*], platypus [ *Ornithorhynchus anatinus*], koala [ *Phascolarctos cinereus*], Tasmanian devil [ *Sarcophilus harrisii*], wombat [ *Vombatus ursinus*]) ( Table 1) and default settings. SAAGA v0.7.9 ( https://github.com/slimsuite/saaga.git) was used to map annotated proteins onto a combined dataset of SwissProt ( Edwards and Palopoli 2015, UniProt 2023) and Quest for Orthologues reference proteomes ( Nevers et al. 2022) to add descriptions and extract the longest isoform per gene for completeness estimation using BUSCO v.5.4.3 (RRID:SCR_015008) in protein mode against the vertebrata_obd10 (n:3354) and mammalia_obd10 lineages (n:9226) ( Simao et al. 2015).

**Table 1.** Assemblies used for GeMoMa annotations.

Common name	Scientific name	Assembly ID	Reference
Cow	*Bos taurus*	ARS-UCD1.2	( Rosen et al. 2020)
Human	*Homo sapiens*	GRCh38.p13	
Opossum	*Monodelphis domestica*	ASM229v1	( Mikkelsen et al. 2007)
Mouse	*Mus musculus*	GRCm39	
Tammar wallaby	*Notamacropus eugenii*	Meug_1.0	( Renfree et al. 2011)
Platypus	*Ornithorhynchus anatinus*	mOrnAna1.p.v.a	( Zhou et al. 2021)
Koala	*Phascolarctos cinereus*	phaCin_unsw_v4.1	( Johnson et al. 2018)
Tasmanian devil	*Sarcophilus harrisii*	mSarHar1.11	( Stammnitz et al. 2023)
Wombat	*Vombatus ursinus*	bare-nosed_wombat_genome_assembly	

The ‘genestats’ script ( https://github.com/darencard/GenomeAnnotation) was used to obtain the average number of exons and introns and the average exon and intron length.

## Results

### Genome assembly

Sequencing generated 185M reads of short read data and 149M reads of 10× Genomics data. Genome assembly with Supernova estimated a 3.79 Gb genome size (46.88X raw coverage) and assembled a 3.57 Gb genome in 38,249 scaffolds (scaffold N50=2.77 Mb) ( Silver 2024). Following Diploidocus cleanup, there were 27,408 primary scaffolds (3.46 Gb) with 1,681 alternative scaffolds (3.01 Gb). Telociraptor made five inversions and trimmed one contig. Contamination removal filtered 786 scaffolds (2.00 Mb) from each haplotype. This gave a final genome size of 3.46 Gb with 26,623 scaffolds ( Table 2). The genome size is comparable to that of other marsupial genomes, including that of the closely related woylie ( *Bettongia penicillate ogilbyi*) ( Haouchar et al. 2016, Peel et al. 2021). BUSCO completeness of the final genome was 92.2% for mammalia_odb10 and over 96.9% for vertebrata_odb10 (96.8% for haplotype two) ( Table 2). Whilst the BUSCO scores suggest a highly complete genome, the use of long read data such as PacBio or Oxford Nanopore would assist in increasing the contiguity of the assembly. Additionally, 53.08% of the genome was identified as repeats, which is similar to other marsupials, including the closely related woylie (53.05%) ( Peel et al. 2021) ( Table 3).

**Table 2.** Genome assembly statistics of the eastern bettong ( *Bettongia gaimardi)* bettong.v1.2hap1 assembly.

Metric	
Assembly size (Gb)	3.46
Number of contigs	91,460
Contig N50 (kb)	87.36
Contig N90 (kb)	64.48
Contig L50	11,569
Contig L90	17,470
Longest contig (kb)	956.31
GC content (%)	38.65
Number of Scaffolds	26,623
Scaffold N50 (Mb)	2.93
Scaffold N90 (Mb)	2.21
Scaffold L50	358
Scaffold L90	524
Longest Scaffold (Mb)	15.08
Complete vertebrata_odb10 BUSCOs	96.9% (Single copy: 92.9%, Duplicated: 4.0%)
Fragmented vertebrata_odb10 BUSCOs	2.0%
Missing vertebrata_odb10 BUSCOs	1.1%
Complete mammalia_odb10 BUSCOs	92.2% (Single copy: 89.4%, Duplicated: 2.8%)
Fragmented mammalia_odb10 BUSCOs	1.8%
Missing mammalia_odb10 BUSCOs	6.0%
Gaps (%)	1.44

**Table 3.** Classification of repeat elements of the eastern bettong ( *Bettongia gaimardi*) genome assembly.

Repeat element	Number of elements	% of sequence
SINEs	2,091,730	8.91
ALUs	19,968	0.10
MIRs	2,069,105	8.08
LINES	3,10,0486	33.21
LINE1	1,049,710	19.2
LINE2	1,146,482	7.49
L3/CR1	563,684	3.09
LTR elements	74,485	0.8
ERVL	15,049	0.18
ERV Class I	23,496	0.23
ERV Class II	23,617	0.28
DNA elements	761,393	2.67
*hAT*- *Charlie*	154,968	0.72
TcMar-Tigger	41,111	0.23
Unclassified	1,179,121	7.5
Total interspersed repeats	1,836,063,741bp	53.08
Small RNA	653	0
Satellites	23,609	0.13

### Genome annotation

Genome annotation with GeMoMa predicted 36,068 and 36,015 protein coding genes for haplotype one and two, respectively, which is a large over-estimation with other marsupials having around 20,000 protein coding genes ( Johnson et al. 2018, Brandies et al. 2020). In the future, generating transcriptomes for a variety of eastern bettong tissues would likely improve the accuracy of the annotation. The annotation is highly complete with 94.1% of mammalian protein BUSCOs complete ( Table 4). The average protein length was 384.3 and 384.7 amino acids for haplotype one and two, respectively, with an average of 6.52 exons per gene ( Table 4). On average, predicted proteins were 87.8% the length of their best SwissProt/QFO hit, suggesting some fragmentation of the annotation, which might be inflating the numbers of annotated genes.

**Table 4.** Statistics of the annotation of the eastern bettong ( *Bettongia gaimardi*).

Metrics	
Annotation	
Complete vertebrata_odb10 BUSCOs	97.3% (Single copy: 93.9%, Duplicated: 3.4%)
Fragmented vertebrata_odb10 BUSCOs	1.6%
Missing vertebrata_odb10 BUSCOs	1.1%
Complete mammalia_odb10 BUSCOs	94.1% (Single copy: 91.5%, Duplicated: 2.6%)
Fragmented mammalia_odb10 BUSCOs	1.7%
Missing mammalia_odb10 BUSCOs	4.2%
Average number of exons per gene	6.52
Average Protein Length (aa)	384.3/384.7 haplotype 1/haplotype 2

### Ethical considerations

Samples were collected under Australian National University Animal Experimentation Ethics Committee ethics protocol A2011/017 (Approved 2011, expired Dec 2014). The sample was collected in during trapping in 2014.

## Data Availability

The raw data are publicly available through the Bioplatforms Australia Oz Mammals Genomes: https://data.bioplatforms.com/organization/bpa-omg . The assembled and annotated genome herein is hosted on the Australasian Genomes site ( https://awgg-lab.github.io/australasiangenomes/) in addition to NCBI. Raw genome sequences are available on: NCBI’s Short Read Archive (SRA): Raw DNA data for generation of genome. SRX26311185 and SRX26311186 ( https://www.ncbi.nlm.nih.gov/sra/PRJNA1095660) ( Silver et al. 2024). The data produced as part of this study are stored on NCBI under BioProjects PRJNA1095660 ( Silver et al. 2024). Databases of molecular data on the NCBI Web site include such examples as nucleotide sequences (GenBank), protein sequences, macromolecular structures, molecular variation, gene expression, and mapping data. They are designed to provide and encourage access within the scientific community to sources of current and comprehensive information. Therefore, NCBI itself places no restrictions on the use or distribution of the data contained therein. Figshare: ARRIVE checklist for A reference genome for the eastern bettong (Bettongia gaimardi), DOI: https://doi.org/10.6084/m9.figshare.27144360.v1 ( Silver 2024). The project contains the following reporting guidelines:
• Author Checklist – ARRIVE Data are available under the terms of the Creative Commons Attribution 4.0 International license (CC-BY 4.0).
