# Supplementary material for: A reference genome for the eastern bettong ( Bettongia gaimardi)
Source: F1000Res. 2025 Jan 27;13:1544. Originally published 2024 Dec 20. [Version 2] doi: 10.12688/f1000research.157851.2 (PMC11733418; doi:10.12688/f1000research.157851.2)
Supplement: Peer review history

# Reviewer report: Reviewer response for version 2

**DOI:** 10.5256/f1000research.177228.r362834

**Published:** 2025-01-28

**Authors:** Németh Attila

**Affiliations:** 1 University of Debrecen, Debrecen, Hungary

Dear Author(s),

 I accept the authors' responses. I have no further comments.

 Best wishes.

Are the datasets clearly presented in a usable and accessible format, and the assembly and annotation available in an appropriate subject-specific repository?

Partly

Are sufficient details of the sequencing and extraction, software used, and materials provided to allow replication by others?

Yes

Are the rationale for sequencing the genome and the species significance clearly described?

Yes

Are the protocols appropriate and is the work technically sound?

Partly

Reviewer Expertise:

molecular phylogentics, phylogeography, (molecular) taxonomy, conservation genetics

I confirm that I have read this submission and believe that I have an appropriate level of expertise to confirm that it is of an acceptable scientific standard.

# Reviewer report: Reviewer response for version 1

**DOI:** 10.5256/f1000research.173363.r351906

**Published:** 2025-01-17

**Authors:** Németh Attila

**Affiliations:** 1 University of Debrecen, Debrecen, Hungary

Dear Editor(s),

 Dear Authors,

 I am sending below my comments on Manuscript, entitled "A reference genome for the eastern bettong ( *Bettongia gaimardi*)," which was submitted for publication in the journal F1000Research.

 I find the article important, of scientific value and interest, because it is the first to provide information on the genome of an animal species, a reference genome, as the authors say, for which we have no such information.

 Nevertheless, some concerns have been raised that make me unsure whether the genomic data provided can be considered a true reference genome.

- Why was the reference genome based on a sample from only one individual? It is not an endangered species, so what was the reasoning behind this choice?
- Why were only short reads performed? The length of repetitive regions is typically much greater than that of short reads, which raises questions about the methodology used.
-  How accurate do the authors estimate their chosen method is?

Although I am not a real expert on genome assembly, I recommend that the authors add a brief section in the manuscript discussing the limitations of their findings. This section could explain the rationale behind their decision to work with a single individual and the reasons for choosing this particular method, such as the use of short reads.

 I also think it is important to briefly discuss what the authors think might explain why this animal has one and a half times as many protein-coding regions as the known marsupial genome. Could there be a biological or evolutionary reason for this?

 Overall, I believe the manuscript is valuable and I support its indexing. While I am uncertain if the results can truly be classified as a reference genome, I am convinced that indexing them will greatly benefit the scientific community. At the same time, I feel that the suggested enhancements could significantly improve the quality of the manuscript and ensure the correct perception and subsequent further use of the results.

 Best regards,

Are the datasets clearly presented in a usable and accessible format, and the assembly and annotation available in an appropriate subject-specific repository?

Partly

Are sufficient details of the sequencing and extraction, software used, and materials provided to allow replication by others?

Yes

Are the rationale for sequencing the genome and the species significance clearly described?

Yes

Are the protocols appropriate and is the work technically sound?

Partly

Reviewer Expertise:

molecular phylogentics, phylogeography, (molecular) taxonomy, conservation genetics

I confirm that I have read this submission and believe that I have an appropriate level of expertise to confirm that it is of an acceptable scientific standard, however I have significant reservations, as outlined above.

# Response

**Published:** 2025-01-19

**Authors:** Luke Silver

**Affiliations:** The University of Sydney, Sydney, New South Wales, Australia

1. Why was the reference genome based on a sample from only one individual? It is not an endangered species, so what was the reasoning behind this choice?
**Response: **A single individual was used as this is still the current best practice in wildlife for generating reference assemblies, we agree that in the near term future it will become more feasible to generate a pangenome for wildlife species to truly capture the entire variation of a species.
2. Why were only short reads performed? The length of repetitive regions is typically much greater than that of short reads, which raises questions about the methodology used.
**Response: **The authors agree that long read sequencing would be more preferable to cover the entire length of repetitive regions, however, at the time sequencing was undertaken (~2017) short reads and 10x were the technologies most accessible to conservation researchers.
3.  How accurate do the authors estimate their chosen method is?
**​​​​​​​Response: **Based of the similarity of the size of the genome assembly and repeat content we can be confident that the genome is highly complete. Additionally our BUSCO analysis suggests a highly complete genome assembly (BUSCO completeness >95%). We agree that utilising long read sequencing would result in an increase in contiguity of the assembly.
4. Although I am not a real expert on genome assembly, I recommend that the authors add a brief section in the manuscript discussing the limitations of their findings. This section could explain the rationale behind their decision to work with a single individual and the reasons for choosing this particular method, such as the use of short reads.
**​​​​​​​Response: **We have added a sentence into the results of the improvements that could be realised with the use of a long read technology. “Whilst the BUSCO scores suggest a highly complete genome, the use of long read data such as PacBio or Oxford Nanopore would assist in increasing the contiguity of the assembly”
5. I also think it is important to briefly discuss what the authors think might explain why this animal has one and a half times as many protein-coding regions as the known marsupial genome. Could there be a biological or evolutionary reason for this?
**​​​​​​​Response: **The increase in the number of protein coding genes could be due to the unavailability of transcriptome data for the species. Which would provide increased support for which of the predicted genes are actually expressed in the species. We have added a sentence in the results stating this. “In the future, generating transcriptomes for a variety of eastern bettong tissues would likely improve the accuracy of the annotation.”

# Reviewer report: Reviewer response for version 1

**DOI:** 10.5256/f1000research.173363.r351899

**Published:** 2025-01-14

**Authors:** Jonathan J Hughes

**Affiliations:** 1 University of California, Riverside, USA

The authors describe a whole-genome assembly generated with 10X sequencing for the eastern bettong, an ecologically important marsupial of conservation concern.  They report their protocols for extraction, sequencing, QC, and annotation. The manuscript is clearly written and the methodology is both appropriate and reasonably detailed. I have a few small concerns that could be addressed:

1. State the parameters used when running any given software. If only default parameters were used for all software, then state that.
2. In Table 2, use commas consistently for reporting large numbers.
3. The number of scaffolds reported in the results (26,663) is different to that reported in Table 2 (26,623).
4. I have been able to locate most of the raw data and the assembled genome through NCBI, but the genome does not appear to be currently hosted on the Australian Genomes website as stated.

Are the datasets clearly presented in a usable and accessible format, and the assembly and annotation available in an appropriate subject-specific repository?

Yes

Are sufficient details of the sequencing and extraction, software used, and materials provided to allow replication by others?

Partly

Are the rationale for sequencing the genome and the species significance clearly described?

Yes

Are the protocols appropriate and is the work technically sound?

Yes

Reviewer Expertise:

Genomics, phylogenetics, mammalogy

I confirm that I have read this submission and believe that I have an appropriate level of expertise to confirm that it is of an acceptable scientific standard.

# Response

**Published:** 2025-01-19

**Authors:** Luke Silver

**Affiliations:** The University of Sydney, Sydney, New South Wales, Australia

1. State the parameters used when running any given software. If only default parameters were used for all software, then state that.
  We have stated in the methods the settings used for running the software
2. In Table 2, use commas consistently for reporting large numbers.
  We have used comma’s consistently throughout the manuscript
3. The number of scaffolds reported in the results (26,663) is different to that reported in Table 2 (26,623).
  We have updated the results to correctly report 26,623 as the number of scaffolds
4. I have been able to locate most of the raw data and the assembled genome through NCBI, but the genome does not appear to be currently hosted on the Australian Genomes website as stated.
  We have uploaded the genome and annotation onto the Australian Genomes website

# Reviewer report: Reviewer response for version 1

**DOI:** 10.5256/f1000research.173363.r351903

**Published:** 2025-01-08

**Authors:** Naoki Osada

**Affiliations:** 1 Hokkaido University, Sapporo, Japan

This manuscript presents the results of whole-genome sequencing and assembly of Bettongia gaimardi. The authors conducted genome assembly,

 quality evaluation, and annotation, including repetitive sequence annotation. I have verified that the assembled genome sequence and raw

 data are publicly available. Overall, this dataset is valuable for future research, and I have only some minor comments:

  

1. In the abstract, showing scaffold N50 would be helpful.
2. In the Introduction section, the phrase “10x short reads” might be misleading, as it could imply a 10-fold coverage of short reads. I suggest using “10x Genomics short reads” or a similar term for clarity.
3. In the Method section, the sentence "Further sequencing using ... was sequenced with 2 lanes ..." requires rephrasing for better readability.
4. It would be helpful if the authors provided additional details about the amount of sequencing data obtained, such as the number of reads or total bases.
5. I assume the authors used default settings for running the software, but it should be stated in the Method section.
6. In the Data availability section, the first sentence would be to: “The raw data are …”.

Are the datasets clearly presented in a usable and accessible format, and the assembly and annotation available in an appropriate subject-specific repository?

Yes

Are sufficient details of the sequencing and extraction, software used, and materials provided to allow replication by others?

Partly

Are the rationale for sequencing the genome and the species significance clearly described?

Yes

Are the protocols appropriate and is the work technically sound?

Yes

Reviewer Expertise:

Population genetics, molecular evolution, genomics

I confirm that I have read this submission and believe that I have an appropriate level of expertise to confirm that it is of an acceptable scientific standard.

# Response

**Published:** 2025-01-19

**Authors:** Luke Silver

**Affiliations:** The University of Sydney, Sydney, New South Wales, Australia

**Reviewer Comment:** In the abstract, showing scaffold N50 would be helpful.

**Author Response:** We have added in the scaffold N50 value to the abstract

**Reviewer Comment: **In the Introduction section, the phrase “10x short reads” might be misleading, as it could imply a 10-fold coverage of short reads. I suggest using “10x Genomics short reads” or a similar term for clarity.

**Author Response: **We have rephrased as suggested

**Reviewer Comment: **In the Method section, the sentence "Further sequencing using ... was sequenced with 2 lanes ..." requires rephrasing for better readability.

**Author Response: **We have rephrased the sentence to “Additional sequencing using the 10X Chromium Genomics library prep with >50 kb size selection was performed using 2 lanes of HiSeq Xten (150 bp PE) (Illumina).” for clarity

**Reviewer Comment: **It would be helpful if the authors provided additional details about the amount of sequencing data obtained, such as the number of reads or total bases

**Author Response: **We have added the amount of reads produced in the first sentence of the results “Sequencing generated 185M reads of short read data and 149M reads of 10x Genomics data”

**Reviewer Comment: **I assume the authors used default settings for running the software, but it should be stated in the Method section.

**Author Response: **We have stated in the methods the settings used for running the software

**Reviewer Comment: **In the Data availability section, the first sentence would be to: “The raw data are …”.

**Author Response: **We have updated the data availability section as suggested
